# Genome Analysis and Description of Three Novel Diazotrophs *Geomonas* Species Isolated From Paddy Soils

**DOI:** 10.3389/fmicb.2021.801462

**Published:** 2022-02-07

**Authors:** Guo-Hong Liu, Shang Yang, Rong Tang, Cheng-Jie Xie, Shun-Gui Zhou

**Affiliations:** ^1^Agricultural Bio-Resources Research Institute, Fujian Academy of Agricultural Sciences, Fuzhou, China; ^2^Fujian Provincial Key Laboratory of Soil Environmental Health and Regulation, College of Resources and Environment, Fujian Agriculture and Forestry University, Fuzhou, China

**Keywords:** *Geomonas oryzisoli*, *Geomonas subterranea*, *Geomonas nitrogeniifigens*, nitrogen fixation activity, iron reduction, paddy soil, genome analysis

## Abstract

Five strictly anaerobic strains, designated RG2^T^, RG3, RG10^T^, RF4^T^, and RG29, were isolated from paddy soils in China. Strains RG2^T^, RF4^T^, RG10^T^, RG3, and RG29 grew at temperatures ranging 5–42°C and pH ranging 5.5–8.5. Strains RG2^T^, RF4^T^, RG3, and RG29 could tolerate NaCl up to 0–0.7% (w/v) while strain RG10^T^ could tolerate NaCl up to 0–0.8% (w/v). The isolated strains showed the highest 16S rRNA gene sequence similarities to the type strains of *Geomonas terrae* Red111^T^ and *Geomonas paludis* Red736^T^. In phylogenetic (based on 16S rRNA gene sequence) and phylogenomic trees, strains clustered with the members of the genus *Geomonas.* Menaquinone-8 was the predominant quinone present in all strains. The major fatty acid profiles of all strains were C_15:1_ ω6*c*, C_16:0_, iso-C_15:0_, and Summed Feature 3. The digital DNA–DNA hybridization (dDDH) and average nucleotide identity (ANI) values between the isolated strains and the closely related *Geomonas* species were lower than the cutoff value (ANI 95–96% and dDDH 70%) for prokaryotic species delineation. Based on physiological, biochemical, and chemotaxonomic properties, strains RG2^T^, RG10^T^, and RF4^T^ could easily be differentiated with the members of the genus *Geomonas*. Additionally, all the isolated strains possessed *nifHDK* clusters and catalytic compartments of nitrogenase. Based on the above results, the isolated five strains represent three novel species of the genus *Geomonas*, for which the names *Geomonas oryzisoli* sp. nov., *Geomonas subterranea* sp. nov., and *Geomonas nitrogeniifigens* sp. nov. are proposed. The type strains are RG10^T^ (= GDMCC1.2537^T^ = KCTC 26318^T^), RG2^T^ (= GDMCC1.2536^T^ = KCTC 25317^T^), and RF4^T^ (= GDMCC 1.2547^T^ = KCTC 25316^T^).

## Introduction

The genus *Geomonas* was established by Xu et al. (2019). Members of the genus *Geomonas* are strictly anaerobic, rod-shaped, and capable of reducing ferric iron to ferrous iron and form reddish colonies due to the presence of *c*-type cytochromes. At the time of writing, there were nine validly published *Geomonas* species^1^ that were isolated from anoxic habitats, such as paddy soils and forest soils (Xu et al., 2019; Itoh et al., 2021).

Biological nitrogen fixation (BNF) is an essential chemical process that involves nitrogen-fixing bacteria, and these bacteria have the potential to minimize the use of N fertilizers in agriculture and reduce the negative impact of nitrogen fertilizers on the environment (Soumare et al., 2020). It has been confirmed that a large amount of nitrogen fertilizer utilization could inhibit and decrease the diversity and ability of nitrogen-fixing bacteria (Feng et al., 2018; Lin et al., 2018; Fan et al., 2019). The dominant diazotrophs in paddy soils were members of the *Geobacteraceae* family with iron reduction abilities (Masuda et al., 2017). In the present study, we isolated five (designated, RG2^T^, RG3, RG10^T^, RF4^T^, and RG29) nitrogen-fixing and iron-reducing strains from paddy soil samples obtained from a greenhouse in Fujian Province, China, where no fertilizer was administered for an extended period. The isolated five strains showed low 16S rRNA gene sequence similarity to members of the genus *Geomonas*; hence, the current investigation was carried out to determine their taxonomic position.

## Materials and Methods

### Isolation and Maintenance

Paddy soil samples were collected from a rice greenhouse belonging to Rice Research Institute, Fujian Academy of Agricultural Sciences, Fuzhou City, Fujian Province, China (N26.1076°, E119.3014°) in January 2021. All strains were isolated based on the soil slurry incubation method according to the description by Xu et al. (2019). Briefly, 10 g fresh paddy soil samples were added aseptically into 120 ml bottles containing 90 ml autoclaved oxygen-free water and shaken in the incubator for half an hour with a speed of 100 rpm at 30°C. The anaerobic and sterile water was obtained by inflating with N_2_:CO_2_ (80:20, v/v) gas mixture for at least 1 h and then autoclaved. Under sterile and oxygen-free conditions, soil suspensions were serially diluted in oxygen-free water at a concentration ranging 10^–1^ to 10^–3^. Then, 200 μl soil suspension from each dilution was spread on R2A agar plates (Hopebio, Qingdao, China) containing 40 mM disodium fumarate (Macklin, Shanghai, China) (modified R2A medium). The plates were then incubated at 30°C for 10 days in Whitley DG250 Anaerobic Workstation (Don Whitley Scientific, Bingley, United Kingdom). The red colonies were picked and purified, repeatedly re-streaked on modified R2A medium, and incubated anaerobically at 30°C for 3 days until pure single colonies were obtained. The purified strains were preserved at −80°C in modified R2A broth supplemented with 10% dimethyl sulfoxide (DMSO) and stored at −80°C. In addition, the reference strains *Geomonas terrae* MCCC 1K04029^T^, *Geomonas paludis* MCCC 1K03950^T^, and *Geomonas oryzae* MCCC 1K03691^T^ (obtained from Marine Culture Collection of China through strains exchange) were cultured on the same medium mentioned as above.

### Microscopic, Physiological, and Biochemical Characteristics

Gram staining was conducted using the KOH method (Gregersen, 1978). Catalase activity was determined by investigating bubble production with 3% (v/v) H_2_O_2_. Oxidase activity was determined using 1% (v/v) tetramethyl-*p*-phenylenediamine (Kovacs, 1956). Cell morphology was observed under a transmission electron microscope (Hitachi, Tokyo, Japan) with negative staining by 1% phosphotungstic acid. The temperature range for growth on the modified R2A agar plate was determined at different temperatures (5, 10, 12, 15, 20, 25, 30, 33, 35, 37, 40, and 42°C) for 5–14 days. The pH range (5.0–9.0 at an interval of 0.5) for growth on modified R2A medium was determined at 30°C for 7 days. The pH of the basal medium was adjusted using the buffer system, as described by Narsing Rao et al. (2020). The effect of NaCl concentration on growth was determined on modified R2A medium supplemented with 0–1.0% (w/v) NaCl. Other enzymatic activities were examined using the API-ZYM microbiological identification system (bioMerieux, Marcy-l’Étoile, France) by following the manufacturer’s instructions. The sensitivity of antibiotics such as clindamycin (2 μg), streptomycin (10 μg), vibramycin (30 μg), erythromycin (15 μg), gentamicin (120 μg), rifampicin (5 μg), vancomycin (30 μg), azithromycin (15 μg), penicillin G (10 μg), cefazolin (30 μg), ampicillin (10 μg), chloromycetin (30 μg), tetracycline (30 μg), oxacillin (1 μg), amikacin (30 μg), polymyxin B (300 IU), gentamicin (10 μg), kanamycin (30 μg), and neomycin (30 μg) was tested.

### 16S rRNA Gene Sequencing and Phylogenetic Analysis

Genomic DNA was extracted using the TIANamp Bacterial DNA kit (TIANGEN, Beijing, China) according to the manufacturer’s instruction. PCR amplification was performed as described by Dong et al. (2019). Pairwise 16S rRNA gene sequence similarities among the isolated five strains and against other type strains were calculated using the EzBioCloud platform (Yoon et al., 2017a). For phylogenetic analysis, 16S rRNA gene sequences of the closely related type strains were downloaded from the EzBioCloud platform. Phylogenetic trees were reconstructed based on the neighbor-joining (NJ) (Saitou and Nei, 1987), maximum likelihood (ML) (Felsenstein, 1981), and maximum-parsimony (Fitch, 1971) methods with the Kimura two-parameter model (Kimura, 1980) and 1,000 bootstrap replications (Felsenstein, 1985) using MEGA version X (Kumar et al., 2018).

### Chemotaxonomic Characterization

For fatty acid detection, the biomass of the isolated five strains and their related type strains was collected from cultures grown for 3 days at 30°*C*. The cellular fatty acids in the cell walls were extracted and analyzed according to the standard protocol of the Microbial Identification System (MIDI) tested by gas chromatography (model 7890, Agilent) (Sasser, 2001). The biomass of the isolated five strains for menaquinone analysis was collected from cultures grown for 7 days at 30°C in modified R2A broth and then analyzed as described by Collins et al. (1977) using reverse-phase HPLC (Kroppenstedt, 1982).

### Genome Sequencing and Analysis

The complete genomes of strains RG2^T^, RG3, RG10^T^, RF4^T^, and RG29 were sequenced using the Nanopore PromethION platform and Illumina NovaSeq PE150 at the Novogene Bioinformatics Technology Company, Ltd. (Beijing, China). A library was reconstructed with the Nanopore PromethION platform (10 kb) and Illumina NovaSeq PE150 (350 bp). Unicycler software (Wick et al., 2017) was used to combine PE150 and Nanopore data, screen the chromosome and plasmid sequence, and assemble the chromosome sequence into a circular genome. Genome quality was estimated by CheckM (Parks et al., 2015). Transfer RNA (tRNA) genes were predicted with tRNAscan-SE version 1.3.1 (Lowe and Eddy, 1997). Ribosomal RNA (rRNA) genes were analyzed with rRNAmmer (Lagesen et al., 2007). Genomic relatedness was estimated based on the average nucleotide identity (ANI) and digital DNA–DNA hybridization (dDDH), which were calculated using the EzBioCloud platform (Yoon et al., 2017b) and Genome-to-Genome Distance Calculator version 2.1 (Meier-Kolthoff et al., 2013), respectively. The phylogenomic tree was reconstructed using the Anvi’o tool (Eren et al., 2015). 38 marker genes of ribosomal proteins (L1, L2, L13, L14, L16, L17, L18p, L19, L20, L21p, L22, L23, L24, L27, L27A, L28, L29, L3, L32p, L35p, L4, L5, L6, L9 C, S10, S11, S13, S15, S16, S17, S19, S2, S3 C, S6, S7, S8, S9, and S20p) were extracted and aligned using MUSCLE (Edgar, 2004) followed by generation of a phylogenomic tree using FastTree (Price et al., 2009). The resulting tree was visualized by MEGA version X (Kumar et al., 2018). Functional annotation was performed by KofamKOALA (Aramaki et al., 2020) using the anvi-run-kegg-kofams program (Eren et al., 2015). The genes related to iron oxidation, iron reduction, iron gene regulation, iron storage, iron transport, magnetosome, and siderophore were checked using FeGenie software (Garber et al., 2020).

### Nitrogen Fixation and Electron Donors and Acceptors

Five isolated strains were grown in 100 ml of modified R2A liquid medium at 30°C for 3 days under anaerobic conditions. Late-log-phase cells were harvested by centrifugation (∼3 × 10^8^ cells/ml) and then washed three times using sterilized ammonium-free MFM (L^–1^: including 2.0 g KHCO_3_, 0.02 g MgSO_4_ 7H_2_O, 0.3 g KH_2_PO_4_, 0.1 g MgCl_2_⋅6H_2_O, 0.08 g CaCl_2_⋅2H_2_O, 0.6 g NaCl, 9.52 g HEPES, 20 mM acetate, 20 mM disodium fumarate), 10.0 ml vitamin stock solution (L^–1^, 2 mg biotin, 2 mg folic acid, 10 mg pyridoxine-HCl, 5 mg thiamine-HCl, 5 mg nicotinic acid, 5 mg aminobenzoic acid, 5 mg Ca-pantothenate, 0.01 mg vitamin B12, and 5 mg lipoic acid), and 10.0 ml mineral stock solution (L^–1^, 12.8 g nitrilotriacetic acid, 1.35 g FeCl_3_⋅6H_2_O, 0.1 g MnCl_2_⋅4H_2_O, 0.024 g CoCl_2_⋅6H_2_O, 0.1 g CaCl_2_⋅2H_2_O, 0.1 g ZnCl_2_, 0.025 g CuCl_2_⋅2H_2_O, 0.01 g H_3_BO_3_, 0.024 g Na_2_MoO_4_⋅2H2O, 1 g NaCl, 0.12 g NiCl_2_⋅6H_2_O, 4 mg Na_2_SeO_3_⋅5H_2_O, 4 mg Na_2_WO_4_, and pH 6.5) liquid medium under sterile conditions. The washed cells were resuspended with 30 ml volumes of sterilized ammonium-free medium in a 100-ml bottle and incubated at 30°C without shaking after being sealed under mixed gas He:C_2_H_2_ (90:10, v/v). The negative control was set up using pure He gas in replacement of He/C_2_H_2_ gas. Nitrogen fixation activity was measured by the traditional acetylene reduction activity (ARA) method based on C_2_H_2_ reduction into C_2_H_4_ by nitrogenase (Postgate, 1972). C_2_H_4_ production in the gaseous phase was measured by gas chromatography equipped with a fused-silica column (Porapak; Hychrom) as described by Nakajima et al. (2012). Cell densities were determined by direct cell counting using a hemocytometer according to the manufacturer’s instructions. The PolF/PolR primer set (Poly et al., 2001) was used to amplify the *nifH* gene, and PCR amplification was carried out as described by Lee et al. (2019).

Electron donor and acceptor utilization tests were performed using 10 ml of degassed modified freshwater medium (MFM) in a 20 ml serum tube under a N_2_/CO_2_ (4:1, v/v) atmosphere at 30°C without shaking. Acetate (10 mM) was used as an electron donor for all electron acceptor tests, while Fe (III)-NTA (5 mM) was used as an electron acceptor for all electron donor tests. The utilized electron donors were determined according to cell growth. The utilization of electron acceptors of Fe (III) compounds was determined by the color change from reddish-brown/green to colorless, whereas those of nitrate, fumarate, and sulfur were determined by cell growth. Fermentation growth in the absence of electron acceptors was evaluated with fumarate, lactate, glycerol, pyruvate, malate, and ethanol by checking the optical density at 600 nm.

### Cytochromes and Ferric Reduction

Cytochrome analysis was prepared from the cells cultured in modified R2A broth. 5 ml cultures were collected and resuspended in 9 ml of 20 mM PIPES buffer (pH 7.0). A dithionite-reduced minus air-oxidized difference spectrum of the whole cells was checked using a UV-2600 (Shimadzu, Japan) spectrophotometer (Lovley et al., 1993). The ferric reduction ability was measured using 10 ml MM (L^–1^: including 2.5 g NaHCO_3_, 0.25 g NH_4_Cl, 0.68 g NaH_2_PO_4_⋅2H_2_O, 0.1 g KCl, 10.0 ml vitamin stock solution, and 10.0 ml mineral stock solution) medium in a 20 ml serum tube supplemented with 10 mM acetate and 20 mM ferrihydrite, under a 80:20 mixture gas of N_2_/CO_2_. The vessel was incubated at 30°C without shaking, and every set of experiments was performed in triplicate. The soluble ferrous concentration was detected immediately using ferrozine buffer as described by Onley et al. (2018). Absorbance was measured using a spectrophotometer (Shimadzu, Japan) at a wavelength of 562 nm. A standard curve was prepared with ferrous ammonium sulfate hexahydrate spanning a dilution concentration range of 0.1–0.8 mM ferrous ions.

## Results and Discussion

### Microscopic, Physiological, and Biochemical Characters

Cells of strains RG2^T^, RG3, RG10^T^, RF4^T^, and RG29 were Gram-stain-negative, non-spore-forming, strictly anaerobic, and motile and produced clear red colonies on a modified R2A agar plate. These characters were similar to *Geomonas* species (Xu et al., 2019; Itoh et al., 2021). Cells of strains were catalase and oxidase negative. TEM images showed rod-shaped cells with peritrichous flagella (Supplementary Figure 1). Strains RG2^T^, RF4^T^, RG10^T^, RG3, and RG29 grew at temperatures 12–42°C (optimum 30°C), 10–40°C (optimum 30–33°C), 16–42°C (optimum 30–33°C), 5–42°C (optimum 30–33°C), and 10–40°C (optimum 30–35°C), respectively. All strains grew at pH ranging 5.5–8.5. Strains RG2^T^, RF4^T^, RG3, and RG29 could tolerate NaCl up to 0–0.7% (w/v) while strain RG10^T^ could tolerate NaCl up to 0–0.8% (w/v). Strains RG2^T^, RG3, RG10^T^, RF4^T^, and RG29 were positive for alkaline phosphatase, but *G. terrae* MCCC 1K04029^T^ and *G. oryzae* S43^T^ (Xu et al., 2019) were negative. Strains RG3, RG29, and *G. paludis* MCCC 1K03950^T^ and *G. oryzae* S43^T^ (Xu et al., 2019) were positive for esterase lipase (C8) but not strains RG2^T^, RG10^T^, RF4^T^, and *G. terrae* MCCC 1K04029^T^. For the antibiotic sensitivity test, five isolated strains RG2^T^, RG3, RG10^T^, RF4^T^, and RG29 were sensitive to clindamycin, chloromycetin, amikacin, kanamycin, rifampin, polymyxin B, vibramycin, tetracycline, and erythromycin, but not to gentamicin, streptomycin, vancomycin, and azithromycin. Strain RG10^T^ was sensitive to oxacillin, but the other four stains were not. Strain RF4^T^ was not sensitive to cefazolin, penicillin G, ampicillin, oxacillin, and neomycin whereas the other four strains were sensitive. Detailed differential features of the isolated five strains and closely related members are listed in Table 1.

**TABLE 1:** Differential characteristics of five isolated strains and closely related members of the genus *Geomonas*.

Characteristics	1	2	3	4	5	6	7	8*
Growth temperature (optimum) (°C)	12–42 (30)	5–42 (30–33)	16–42 (30–33)	10–40 (30–33)	10–40 (30–35)	20–42 (30–33)	10–42 (30–33)	13–42 (30–33)
Growth pH (optimum)	5.5–8.5 (6.0–7.0)	5.5–8.5 (6.5–7.5)	5.5–8.5 (6.5–7.0)	5.5–8.5 (6.0–7.0)	5.5–8.5 (6.0–7.0)	5.5–7.5 (6.0–7.0)	5.5–8.0 (6.0–7.0)	5.5–8.0 (6.0–7.0)
Growth NaCl (%) (optimum)	0–0.7 (0.2–0.3)	0–0.7 (0–0.3)	0–0.8 (0–0.4)	0–0.7 (0.2–0.4)	0–0.7 (0.2–0.4)	0–0.8 (0–0.2)	0–0.7 (0–0.2)	0–0.7 (0–0.2)
**Electron donors**								
Toluene	+	+	+	+	+	−	−	−
Butanol	+	+	+	+	+	−	−	−
Methanol	+	+	+	+	+	−	+	+
Propionate	+	+	+	+	+	+	−	−
**Electron acceptors**								
Fe (III) citrate	+	+	−	+	+	+	−	−
**Enzymatic activities**								
Alkaline phosphatase	+	+	+	+	+	+	−	−
Esterase (C4)	+	-	w	−	+	+	−	+
Esterase lipase (C8)	−	+	−	−	+	+	−	+
Leucine arylamidase	+	+	+	−	+	+	+	+
Absorbance peaks of cytochrome *c* (nm)	424, 524, and 553	424, 524, and 553	424, 524, and 554	425, 523, and 554	425, 523, and 554	424, 524, and 554	424, 524, and 554	424, 524, and 554

1, RG2^T^; 2, RG3; 3, RG10^T^; 4, RF4^T^; 5, RG29; 6, Geomonas paludis MCCC 1K03950^T^; 7, Geomonas terrae MCCC 1K04029^T^; and 8, Geomonas oryzae S43^T^. All strains could use lactate, malate, succinate, and fumarate as electron donors but not phenol and benzaldehyde. All could use nitrate as electron acceptors but sulfur and MnO_2_ not. The ability of acid phosphatase and naphthol-AS-BI-phosphohydrolase for all strains was positive, but lipase (C14), valine arylamidase, cystine arylamidase, trypsin, α-chymotrypsin, α-galactosidase, β-galactosidase, β-glucuronidase, α-glucosidase, β-glucosidase, N-acetyl-β-glucosaminidase, α-mannosidase, and β-fucosidase were negative. +, positive; −, negative; w, weakly positive; *, data from reference Xu et al. (2019).

### 16S rRNA Gene Sequencing and Phylogenetic Analysis

The isolated strains, RG2^T^, RG3, RG10^T^, RF4^T^, and RG29 had 98.5–99.9% 16S rRNA gene sequence similarity to each other. Strains RG2^T^, RG3, and RG29 showed the highest 16S rRNA gene sequence similarities to the type strain of *G. terrae* Red 111^T^ (98.2–98.5%) (Supplementary Table 1). These values were below the threshold (98.65%) for species delineation (Kim et al., 2014) but above the threshold (94.55–95.05%) for genus delineation (Yarza et al., 2014), suggesting that these strains were different at the species level within the genus *Geomonas*. Strains RF4^T^ and RG10^T^ had the highest similarity to the type strain of *G. paludis* Red 736^T^ (99.1–99.3%). Despite the fact that the sequence similarity of RF4^T^ and RG10^T^ was quite high, numerous earlier studies have revealed that two strains with 16S rRNA gene similarity greater than 99.0% were verified as separate species (Tohno et al., 2017; Hahn et al., 2018; Qin et al., 2020). A range of thresholds (98.2–99.0%) was also recommended by Meier-Kolthoff et al. (2013). Therefore, strains RG10^T^ and RF4^T^ might be novel species of the genus *Geomonas*.

**FIGURE 1:**
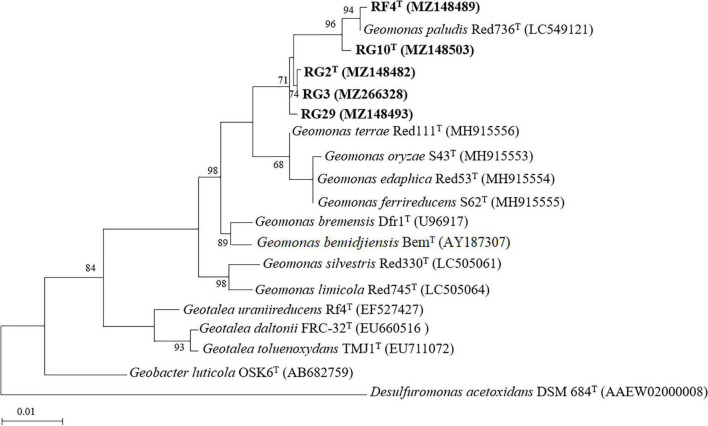
Maximum-likelihood phylogenetic tree based on 16S rRNA gene sequence of five isolated strains and their closely related type strains. Bootstrap values (expressed as percentages of 1,000 replications) greater than 50% are shown at branch points. Bar, 0.01 substitutions per nucleotide position. *Desulfuromonas acetoxidans* DSM 684^T^ is used as an outgroup. Number in the parenthesis is the GenBank accession number.

**FIGURE 2:**
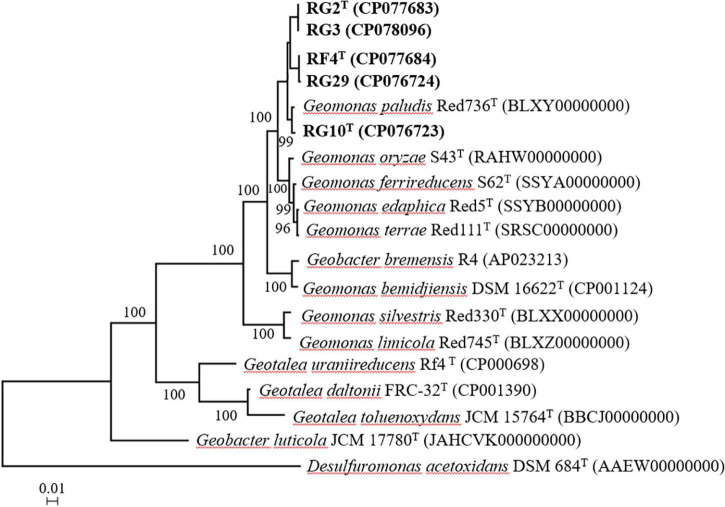
Phylogenomic tree based on the amino acid sequences of 38 ribosomal proteins of five strains and their closely related strains. Bootstrap values (expressed as percentages of 1,000 replications) greater than 50% are shown at branch points. Bar, 0.01 substitutions per nucleotide position. *Desulfuromonas acetoxidans* DSM 684^T^ is used as an outgroup. Number in the parenthesis is the genome accession number.

The maximum likelihood phylogenetic tree based on the 16S rRNA gene sequences showed that strains RG2^T^, RG3, RG10^T^, RF4^T^, and RG29 clade with *Geomonas* members (Figure 1). The phylogenetic position was also confirmed by the trees generated using neighbor-joining (Supplementary Figure 2) and maximum parsimony (Supplementary Figure 3) methods. In the phylogenomic tree, all strains clustered with the members of the genus *Geomonas* (Figure 2).

### Chemotaxonomic Characters

The fatty acid profiles clearly distinguished the isolated five strains RG2^T^, RG3, RG10^T^, RF4^T^, RG29, and their related type strains. All five strains contained C_15:1_ ω6*c*, iso-C_15:0_, C_16:0_, and Summed Feature 3 as major fatty acid profiles, but their proportion was distinguished. Strains RG2^T^, RG3, RG10^T^, RF4^T^, and RG29 had iso-C_15:0_ and Summed Feature 3 levels more than 10% (Table 2), but it was < 10% in *G. paludis* MCCC 1K03950^T^ and *G*. *oryzae* MCCC 1K03691^T^. Fatty acid C_17:1_ ω6*c* presented in strains RG2^T^, RG3, and type strain *G*. *paludis* MCCC 1K03950^T^ was above 5% whereas it was much lower than 5% in other three strains and related type strains *G. terrae* MCCC 1K04029^T^ and *G*. *oryzae* MCCC 1K03691^T^. C_15:1_ ω6*c* was high in strain RG2^T^ and C_16:0_ in RG10^T^; this value was less when compared with *G. paludis* MCCC 1K03950^T^. The iso-C_15:0_ value in *G. terrae* MCCC 1K04029^T^ and *G*. *oryzae* MCCC 1K03691^T^ was higher compared with other strains. Detailed comparative fatty acid profiles of strains RG2^T^, RG3, RG10^T^, RF4^T^, RG29, *G. paludis* MCCC 1K03950^T^, *G. oryzae* MCCC 1K03691^T^, and *G. terrae* MCCC 1K04029^T^ are mentioned in Table 2.

**TABLE 2:** Fatty acid profile of isolated five strains and closely related species of the genus *Geomonas.*

Fatty acids (%)	1	2	3	4	5	6	7	8
anteiso-C_11:0_	1.7	–	–	–	–	–	–	<1
anteiso-C_13:0_	1.6	2.1	1.1	1.6	1.1	<1	<1	<1
anteiso-C_15:0_	<1	–	<1	1.1	<1	1.1	<1	<1
C_10:0_ 2OH	–	1.2	<1	<1	<1	–	–	–
C_13:0_	1.7	1.3	1.1	1.3	1.0	1.6	1.0	1.1
C_14:0_	4.3	**5.3**	**7.1**	**5.3**	**5.1**	3.6	3.7	3.1
C_15:0_ 3OH	4.2	3.3	2.6	3.3	2.8	**7.4**	–	–
**C_15:1_ ω6c**	**14.5**	**9.7**	**7.7**	**10.7**	**9.0**	**21.4**	**5.4**	**12.5**
C_15:1_ ω8*c*	1.1	–	<1	<1	–	<1	–	<1
**C_16:0_**	**8.8**	**11.6**	**13.4**	**12.2**	**10.0**	**14.6**	**9.3**	**8.0**
C_16:0_ 3OH	3.7	4.4	4.7	3.8	4.0	2.4	4.1	2.9
C_16:1_ iso H	3.9	2.9	–	3.1	2.7	2.8	–	3.6
C_16:1_ ω5c	1.5	2.0	1.1	1.2	1.2	<1	<1	<1
C_17:0_	1.4	1.5	<1	3.1	<1	1.9	<1	1.0
C_17:0_ 3OH	2.6	–	1.9	3.1	1.6	2.1	–	2.0
C_17:1_ ω6*c*	**5.3**	**5.0**	3.2	4.7	3.8	**8.1**	1.8	3.6
C_18:1_ ω9*c*	1.3	1.6	1.2	1.2	<1	<1	–	–
C_19:0_	1.2	–	<1	1.3	<1	<1	–	–
iso-C_13:0_ 3OH	1.1	–	–	1.4	1.0	–	–	–
iso-C_13:0_	<1	<1	<1	<1	<1	<1	1.7	1.2
iso-C_14:0_	–	–	–	<1	–	<1	–	<1
**iso-C_15:0_**	**14.9**	**18.0**	**17.1**	**11.8**	**21.1**	**10.6**	**33.8**	**24.8**
iso-C_15:0_ 3OH	3.2	4.1	3.4	3.3	4.1	3.4	**12.3**	**6.7**
iso-C_15:1_ F	<1	<1	1.2	1.3	1.0	<1	2.0	1.4
Summed Feature 1	<1	<1	<1	<1	<1	<1	1.1	<1
Summed Feature 2	1.6	1.9	3.8	1.0	3.5	<1	3.3	<1
**Summed Feature 3**	**13.1**	**16.9**	**18.2**	**16.6**	**18.6**	**7.6**	**10.1**	**9.6**

*1, RG2^T^; 2, RG3; 3, RG10^T^; 4, RF4^T^; 5, RG29; 6, Geomonas paludis MCCC 1K03950^T^; 7, Geomonas terrae MCCC 1K04029^T^; 8, Geomonas oryzae MCCC 1K03691^T^. All data were obtained from this study. Only content > 1% was shown. –, not detected. The values ≥ 5.0% were highlighted in bold. Summed Features are the fatty acids that cannot be separated using the chromatographic conditions chosen. The MIDI system groups these fatty acids together as one feature with a single percentage of the total. Summed Feature 1, iso-C_15:1_ H and/or C_13:0_ 3OH; Summed Feature 2, C_12:0_ aldehyde and/or unknown 10.928; Summed Feature 3: C_16:1_ ω7c and/or C_16:1_ ω6c.*

The menaquinone composition was detected for all five isolated strains in the present study, menaquinone-8 (MK-8) was characterized as the predominant respiratory quinone, and small amounts of MK-7 and MK-9 were also identified for all strains, which was consistent with other type strains of the genus *Geomonas* (Xu et al., 2019; Itoh et al., 2021).

### Genome Analysis

The genome size of strains RG2^T^, RG3, RG10^T^, RF4^T^, and RG29 was ∼4.76–4.99 Mb, containing 4,394, 4,341, 4,181, 4,224, and 4,138 protein-coding genes, respectively (Supplementary Table 2). All five isolated strains had 12 rRNAs including 4 of 5S, 4 of 16S, and 4 of 23S rRNAs (Supplementary Table 2). The 16S rRNA gene of strains RG2^T^, RG3, RG10^T^, RF4^T^, and RG29 obtained genome was similar to the 16S rRNA gene obtained by PCR (Supplementary Table 2). The genomic DNA G + C content of strains RG2^T^, RG3, RG10^T^, RF4^T^, and RG29 was 61.9, 61.9, 61.8, 61.7, and 62.0%, respectively (Supplementary Table 2). The genomic similarity values between the five isolated strains and the type strains of *Geomonas* species were below 96% for ANI and 70% for dDDH. Moreover, the ANI and dDDH values among the five isolated strains were 87.2–99.4% and 32.4–100%, respectively (Supplementary Table 3). Considering the recommended thresholds for species delineation, 96% for ANI, and 70% for dDDH (Goris et al., 2007; Richter and Rosselló-Móra, 2009; Meier-Kolthoff et al., 2013), strains RG2^T^, RG10^T^, and RF4^T^ were suggested to be three different species of genus *Geomonas*.

Functional annotation (Supplementary Table 4) analysis suggests that all strains encode key genes for glycolysis and tricarboxylic acid but only strains RG2^T^ and RG3 encode genes for the pentose phosphate pathway.

Autotrophic prokaryotic representatives play an important role in ecosystems by supplying organic carbon to heterotrophs (Hügler et al., 2005). The Calvin–Benson–Bassham cycle is the most significant autotrophic carbon fixation mechanism that exists today (Bassham et al., 1954; Hügler et al., 2005). Despite its worldwide importance, it is limited to species having a high-energy production from a chemotrophic or phototrophic lifestyle. Microorganisms found in extreme environments, such as high temperatures and anaerobic or acidic conditions, generally use different CO_2_ fixation pathways, and three alternative pathways are currently known: the reductive tricarboxylic acid cycle, the reductive acetyl coenzyme A (CoA) pathway, and the 3-hydroxypropionate cycle (Hügler et al., 2005). The key genes for the Calvin–Benson–Bassham and 3-hydroxypropionate cycle were not noticed in any strains, but the key genes related to the reductive tricarboxylic acid cycle were present in all strains.

BNF is the most common method of converting atmospheric nitrogen gas (N_2_) to ammonia, and all known diazotrophs include at least one of three closely related nitrogenase subtypes: Nif, Vnf, and Anf. The *nifHDK* core set and catalytic compartment of nitrogenase (Dos Santos et al., 2012) were noticed in all strains (Supplementary Tables 2, 4). Furthermore, genes related to dissimilatory nitrate reduction (*narGHI* and *nrfA*) were also noticed in all strains.

Members of the genus *Geomonas* were known for metal reduction (Xu et al., 2019). It has been demonstrated that certain *c*-type cytochromes may form a conductive channel capable of crossing the inner membrane, periplasm, and outer membrane, allowing *Geobacteraceae* species to directly transport electrons from the quinone/quinol pool to metal oxides (Guo et al., 2021). The bacteria develop electrically conductive protein nanowires, known as e pili, which are built from the PilA pilin monomer to perform long-range extracellular electron transfer to metal oxide particles (Lovley and Walker, 2019; Guo et al., 2021). Furthermore, protein components such as CymA, MtrA, MtrB, MtrC, and OmcA have also been reported for Fe(III) oxide reduction (Shi et al., 2012). In the present study, all strains encode genes for iron reduction, iron gene regulation, iron acquisition-iron transport, and siderophore transport (Supplementary Figure 4). The details of the key genes for iron reduction, iron gene regulation, iron acquisition-iron transport, and siderophore transport are mentioned in Supplementary Table 5. All strains harbored *merA* but not *merB*, indicating that they might be susceptible to mercury. Although previous studies indicated that certain *Geomonas* species contained *kaiBC* genes (encoding essential proteins in circadian rhythms) (Wang et al., 2019; Itoh et al., 2021), we did not detect this in our study. Details of all the metabolic potential of the present study strains are mentioned in Supplementary Table 4.

### Nitrogen Fixation and Electron Donors and Acceptors

In all strains, the *nifH* gene was amplified (Supplementary Figure 5A). Nitrogen fixation activity was also estimated based on acetylene reduction activity (ARA). The ARAs of strains RG2^T^, RG3, RG10^T^, RF4^T^, and RG29 were (1.7 ± 0.2) × 10^–6^, (2.1 ± 0.4) × 10^–6^, (1.2 ± 0.7) × 10^–6^, (3.2 ± 0.2) × 10^–6^, and (2.8 ± 0.2) × 10^–6^ nmol C_2_H_4_/h/cell, respectively (Supplementary Figure 5B). Therefore, it suggests that all the isolated five strains could fix nitrogen. Supplementary Figure 5B clearly shows that strain RF4^T^ had the highest nitrogen-fixing ability among all the strains.

The availability of 10 electron donors for the isolated five strains was similar, while electron acceptors were different (Table 1); for example, strains RG2^T^, RG3, RF4^T^, and RG29 could utilize Fe (III) citrate as an electron acceptor but strain RG10^T^ could not. For the fermentation growth, strains RF4^T^, RG2^T^, RG3, RG10^T^, and RG29 showed weak growth using fumarate as a substrate in the absence of electron acceptors, but RF4^T^ and RG29 did not grow with malate and pyruvate. Strain RG10^T^ did not grow with lactate and glycerol. Strain RG3 grew with ethanol but not strain RG2^T^.

### Cytochromes and Ferric Reduction

Five isolated strains possessed ferric reducing ability, which reduced ferrihydrite to ferrous iron in 25 days. Although they had ferric reducing ability, their reduction rates were different (Supplementary Figure 6). Strain RF4^T^ showed the highest iron reduction ability among the five strains.

The spectra of whole cells of all the isolated five strains suggest they could produce *c*-type cytochromes (Supplementary Figure 7). The absorbance peaks of reduced cytochrome *c* of strains RG2^T^, RG3, RG10^T^, RF4^T^, and RG29 were 424, 524, and 553 nm; 424, 524, and 553 nm; 424, 524, and 554 nm; 425, 523, and 554 nm; 425, 523, and 554 nm, respectively, which were quite similar to those of the type strains of *Geomonas* species (Table 1).

### Taxonomic Conclusion

Based on the above results, three strains, RG10^T^, RG2^T^, and RF4^T^ represent three novel species of the genus *Geomonas*, for which the names *Geomonas oryzisoli* sp. nov., *Geomonas subterranea* sp. nov., and *Geomonas nitrogeniifigens* sp. nov. are proposed.

### Description of *Geomonas oryzisoli* sp. nov.

*Geomonas oryzisoli* (o.ry.zi.so’li L. fem. n. *oryza*, rice; L. neut. n. *solum* soli, of the soil; N.L. gen. n. *oryzisoli* of rice root soil).

Cells are Gram-stain-negative, non-spore-forming, motile (with peritrichous flagella), strictly anaerobic, and rod-shaped. Colonies are small, red, and circular. Growth occurs at 16–42°C (optimum 30–33°C), pH 5.5–8.5 (optimum 6.5–7.0), and 0–0.8% (w/v) NaCl (optimum 0–0.4%). Catalase and oxidase tests are negative. With Fe (III)-NTA as an electron acceptor, acetate, toluene, butanol, methanol, lactate, malate, propionate, succinate, and fumarate can be used as electron donors but not phenol and benzaldehyde. With acetate as electron donor, Fe (III)-NTA, ferrihydrite, fumarate, nitrate, and malate can be utilized as electron acceptors, but not Fe (III)-citrate, oxygen, sulfur, and MnO_2_. Weak growth is observed using fumarate, ethanol, malate, and pyruvate as a substrate in the absence of electron acceptors but not with lactate and glycerol. It is positive for alkaline phosphatase, esterase (C4), leucine arylamidase, acid phosphatase, and naphthol-AS-BI-phosphohydrolase but negative for esterase (C4), esterase lipase (C8), lipase (C14), valine arylamidase, cystine arylamidase, trypsin, α-chymotrypsin, α-galactosidase, β-galactosidase, β-glucuronidase, α-glucosidase, β-glucosidase, *N*-acetyl-β-glucosaminidase, α-mannosidase, and β-fucosidase. The genome consists of a dinitrogenase core gene set of *nifHDK*. The major fatty acids (>5%) are C_14:0_, C_15:1_ ω6*c*, C_16:0_, iso-C_15:0_, and Summed Feature 3. MK-8 is the major respiratory quinone. The genomic DNA G + C content is 61.8%.

The type strain, RG10^T^ (= GDMCC1.2537^T^ = KCTC 26318^T^), was isolated from paddy soil collected from the greenhouse in Fujian Province, China. The 16S rRNA gene and whole-genome sequence of strain RG10^T^ have been deposited in the GenBank/ENA/DDBJ under the accession numbers MZ148503 and CP076723, respectively.

### Description of *Geomonas subterranea* sp. nov.

*Geomonas subterranea* (sub.ter.ra’ne.a L. fem. adj. *subterranea*, underground).

Cells are Gram-stain-negative, non-spore-forming, motile (with peritrichous flagella), strictly anaerobic, and rod-shaped. Colonies are small, red, and circular. Growth occurs at 12–42°C (optimum 30°C), pH 5.5–8.5 (optimum 6.0–7.0), and 0–0.7% (w/v) NaCl (optimum 0.2–0.3%). Catalase and oxidase tests are negative. With Fe (III)-NTA as an electron acceptor, acetate, toluene, butanol, methanol, lactate, malate, propionate, succinate, and fumarate can be used as electron donors but not phenol and benzaldehyde. With acetate as electron donor, Fe (III)-NTA, Fe (III)-citrate, ferrihydrite, fumarate, nitrate, and malate can be utilized as electron acceptors, but not oxygen, sulfur, and MnO_2_. Weak growth is observed using fumarate, glycerol, malate, lactate, pyruvate, and fumarate as a substrate in the absence of electron acceptors, but not with ethanol. It is positive for alkaline phosphatase, esterase (C4), leucine arylamidase, acid phosphatase, and naphthol-AS-BI-phosphohydrolase, but negative for esterase lipase (C8), lipase (C14), valine arylamidase, cystine arylamidase, trypsin, α-chymotrypsin, α-galactosidase, β-galactosidase, β-glucuronidase, α-glucosidase, β-glucosidase, *N*-acetyl-β-glucosaminidase, α-mannosidase, and β-fucosidase. The major fatty acids (>5%) are C_15:1_ ω6c, C_16:0_, C_17:1_ ω6*c*, iso-C_15:0_, and Summed Feature 3. The genome consists of a dinitrogenase core gene set of *nifHDK*. MK-8 is the major respiratory quinone. The genomic DNA G + C content is 61.9%.

The type strain, RG2^T^ (= GDMCC1.2536^T^ = KCTC 25317^T^), was isolated from paddy soil collected from the greenhouse in Fujian Province, China. The 16S rRNA gene and whole-genome sequence of strain RG2^T^ have been deposited in the GenBank/ENA/DDBJ under the accession numbers MZ148482 and CP077683, respectively.

### Description of *Geomonas nitrogeniifigens* sp. nov.

*Geomonas nitrogeniifigens* (ni.tro.ge.ni.i’fi.gens N.L. neut. n. *nitrogenium* nitrogen; L. pres. part. *figens* fixing; N.L. part. adj. *nitrogeniifigens*, nitrogen-fixing).

Cells are Gram-stain-negative, non-spore-forming, motile (with peritrichous flagella), strictly anaerobic, and rod-shaped. Colonies are small, red, and circular. Growth occurs at 10–40°C (optimum 30–33°C), pH 5.5–8.5 (optimum pH 6.0–7.0), and 0–0.7% (w/v) NaCl (optimum 0.2–0.4%). Catalase and oxidase are negative. With Fe (III) –NTA as an electron acceptor, acetate, toluene, butanol, methanol, lactate, malate, propionate, succinate, and fumarate can be used as electron donors but not phenol and benzaldehyde. With acetate as electron donor, Fe (III)-citrate, Fe (III)-NTA, ferrihydrite, fumarate, and nitrate can be utilized as electron acceptors, but not malate, oxygen, and sulfur and MnO_2_. Weak growth is observed using fumarate, ethanol, glycerol, and lactate as a substrate in the absence of electron acceptors, but not with malate and pyruvate. It is positive for alkaline phosphatase, acid phosphatase, and naphthol-AS-BI-phosphohydrolase, but negative for esterase (C4), esterase lipase (C8), lipase (C14), leucine arylamidase, valine arylamidase, cystine arylamidase, trypsin, α-chymotrypsin, α-galactosidase, β-galactosidase, β-glucuronidase, α-glucosidase, β-glucosidase, *N*-acetyl-β-glucosaminidase, α-mannosidase, and β-fucosidase. The genome consists of a dinitrogenase core gene set of *nifHDK*. The major fatty acids are C_14:0_, C_15:1_ ω6*c*, C_16:0_, iso-C_15:0_, and Summed Feature 3. MK-8 is the major respiratory quinone. The genomic DNA G + C content is 61.7%.

The type strain, RF4^T^ (= GDMCC 1.2547^T^ = KCTC 25316^T^), was isolated from paddy soil collected from the greenhouse in Fujian Province, China. The 16S rRNA gene and whole-genome sequence of strain RF4^T^ have been deposited in GenBank/ENA/DDBJ under the accession numbers MZ148489 and CP077684, respectively.

## Accession Numbers

The GenBank accession numbers for 16S rRNA gene and genome sequence of strains RG2^T^, RG3, RG10^T^, RF4^T^, and RG29 were MZ148482 and CP077683, MZ266328 and CP078096, MZ148503 and CP076723, MZ148489 and CP077684, and MZ148493 and CP076724, respectively.

## Data Availability Statement

The datasets presented in this study can be found in online repositories. The names of the repository/repositories and accession number(s) can be found below: https://www.ncbi.nlm.nih.gov/nuccore/CP077683.1, https://www.ncbi.nlm.nih.gov/nuccore/CP078096.1, https://www.ncbi.nlm.nih.gov/nuccore/CP076723.1, https://www.ncbi.nlm.nih.gov/nuccore/CP077684.1, and https://www.ncbi.nlm.nih.gov/nuccore/CP076724.1.

## Author Contributions

G-HL and S-GZ designed the research and project outline. SY and RT performed the DNA extraction and chemotaxonomic analysis. C-JX performed the genome analysis. G-HL drafted the manuscript. All authors read and approved the final manuscript.

## Conflict of Interest

The authors declare that the research was conducted in the absence of any commercial or financial relationships that could be construed as a potential conflict of interest.

## Publisher’s Note

All claims expressed in this article are solely those of the authors and do not necessarily represent those of their affiliated organizations, or those of the publisher, the editors and the reviewers. Any product that may be evaluated in this article, or claim that may be made by its manufacturer, is not guaranteed or endorsed by the publisher.

## Footnotes

1
https://lpsn.dsmz.de/genus/geomonas

## Abbreviations

dDDH: digital DNA-DNA hybridization
ANI: average nucleotide identity.
