# Supplementary material for: Genome Analysis and Description of Three Novel Diazotrophs Geomonas Species Isolated From Paddy Soils
Source: Front Microbiol. 2022 Feb 7;12:801462. doi: 10.3389/fmicb.2021.801462 (PMC8859169; doi:10.3389/fmicb.2021.801462)
Supplement: Supplementary file 1 [file Data_Sheet_1.docx]

**Supplementary files**

**Genome analysis and description of three novel diazotrophs *Geomonas* species isolated from paddy soils**

**Guo-Hong Liu^1^, Shang Yang^1,2^, Rong Tang^1,2^, Cheng-Jie Xie^1,2^, Shun-Gui Zhou^2*^**

^1^Agricultural Bio-resources Research Institute, Fujian Academy of Agricultural Sciences, Fuzhou City, Fujian Province, 350003, PR China.

^2^Fujian Provincial Key Laboratory of Soil Environmental Health and Regulation, College of Resources and Environment, Fujian Agriculture and Forestry University, Fuzhou City, Fujian Province, 350002, PR China.

***Authors for correspondence:**

Shun-Gui Zhou

Tel & Fax: +86 591 86397843

E-mail: sgzhou@fafu.edu.cn

**Running title:** Three novel diazotrophic *Geomonas* species

**Table S1.** 16S rRNA gene sequence similarities of strains and their closely related species.

| Strains similarity (%) | RG2^T^ | RG3 | RG10^T^ | RF4^T^ | RG29 | *Geomonas paludis* Red736^T^ | *Geomonas terrae* Red111^T^ |
| --- | --- | --- | --- | --- | --- | --- | --- |
| RG2^T^ | - | 99.7 | 98.3 | 98.0 | 99.6 | 98.2 | 98.1 |
| RG3 | 99.7 | - | 98.2 | 98.3 | 99.5 | 98.3 | 98.3 |
| RG10^T^ | 98.3 | 98.2 | - | 98.8 | 98.4 | 99.0 | 97.3 |
| RF4^T^ | 98.0 | 98.3 | 98.8 | - | 97.7 | 99.9 | 97.4 |
| RG29 | 99.6 | 99.5 | 98.4 | 97.7 | - | 98.1 | 98.5 |

**Table S2**. Genome attributes of five strains isolated from paddy soil.

| **Characteristics** | RG2^T^ | RG3 | RG10^T^ | RF4^T^ | RG29 |
| --- | --- | --- | --- | --- | --- |
| Genome size (bp) | 4991523 | 4991984 | 4846465 | 4829642 | 4761015 |
| Genomic DNA G+C content (%) | 61.9 | 61.9 | 61.8 | 61.7 | 62.0 |
| No. of rRNAs (5S/16S/23S) | 4/4/4 | 4/4/4 | 4/4/4 | 4/4/4 | 4/4/4 |
| No. of tRNAs | 59 | 59 | 58 | 61 | 58 |
| Genome completeness/contamination (%) | 99.3/zero | 99.3/zero | 99.3/zero | 100/0.6 | 100/zero |
| *nif* genes | *nifBHDKJEVX* | *nifBHDKEVX* | *nifBHDKJEVX* | *nifBHDKJEVX* | *nifBHDKJEVX* |
| Similarity between 16S rRNA gene from genome vs 16S rRNA gene derived by PCR (%) | 99.4-99.7 | 99.8 | 99.7 | 98.1 | 100 |
| Genome accession number | CP077683 | CP078096 | CP076723 | CP077684 | CP076724 |

**Table S3.** ANI and dDDH values of five strains and their related species in genus *Geomonas.*

| Strains | RF4^T^ | | RG2^T^ | | RG3 | | RG10^T^ | | RG29 | |
| --- | --- | --- | --- | --- | --- | --- | --- | --- | --- | --- |
|  | dDDH | ANI | dDDH | ANI | dDDH | ANI | dDDH | ANI | dDDH | ANI |
| RG2^T^ | 33.8 | 87.3 | 100 | 100 | **100** | **99.9** | 32.4 | 86.6 | 33.8 | 87.5 |
| RG3 | 33.8 | 87.5 | **100** | **99.9** | 100 | 100 | 32.4 | 86.4 | 33.8 | 87.2 |
| RG10^T^ | 35.3 | 88.1 | 32.4 | 86.6 | 32.4 | 86.4 | 100 | 100 | 35.5 | 88.1 |
| RF4^T^ | 100 | 100 | 33.8 | 87.3 | 33.8 | 87.5 | 35.3 | 88.1 | **95.3** | **99.4** |
| RG29 | **95.3** | **99.4** | 33.8 | 87.5 | 33.8 | 87.2 | 35.5 | 88.1 | 100 | 100 |
| *Geobacter bemidjiensis* Bem^T^ | 23.4 | 79.1 | 23.2 | 79.1 | 23.2 | 79.1 | 23.3 | 79.2 | 23.3 | 79.2 |
| *Geomonas edaphica* Red5^T^ | 24.6 | 81.3 | 25.0 | 81.1 | 25.0 | 81.0 | 24.8 | 81.3 | 24.8 | 81.3 |
| *Geomonas terrae* Red111^T^ | 25.0 | 81.4 | 25.1 | 87.0 | 25.1 | 81.2 | 24.9 | 81.3 | 24.9 | 81.5 |
| *Geomonas paludis* Red736^T^ | 33.5 | 87.2 | 33.2 | 86.9 | 33.2 | 87.0 | **35.4** | **88.0** | 33.6 | 87.4 |
| *Geomonas oryzae* S43^T^ | 25.3 | 81.6 | 25.5 | 81.6 | 25.5 | 81.5 | 25.4 | 81.5 | 25.3 | 81.6 |
| *Geomonas silvestris* Red330^T^ | 21.9 | 77.3 | 21.7 | 77.2 | 21.7 | 77.0 | 21.8 | 77.2 | 21.9 | 77.3 |
| *Geomonas limicola* Red745^T^ | 21.8 | 76.9 | 21.7 | 76.8 | 21.7 | 76.9 | 21.9 | 76.8 | 21.8 | 76.9 |
| *Geomonas ferrireducens* S62^T^ | 25.1 | 81.4 | 25.3 | 81.5 | 25.3 | 81.4 | 24.9 | 81.3 | 25.1 | 81.5 |

| 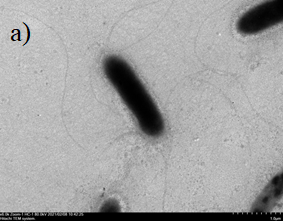 | 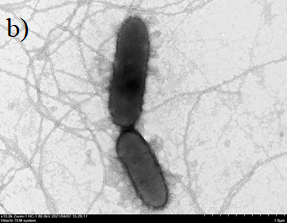 |
| --- | --- |
|  |  |
| 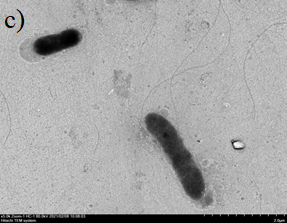 | 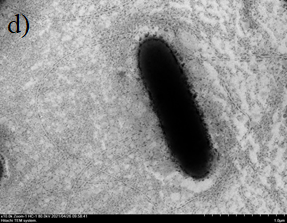 |
|  |  |
| 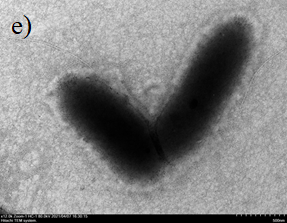 |  |

**Supplementary Figure S1.** Transmission electron micrograph of strains grown on R2A agar supplemented with 40 mM fumarate at 30 °C for 5 days. a) RF4^T^, b) RG29, c) RG2^T^, d) RG3, e) RG10^T^.

**RG29 (MZ148493/1410)**

**RF4^T^ (MZ148489/1389)**

**RG2^T^ (MZ148482/1405)**

**RG3 (MZ266328/1436)**

*Geomonas paludis* Red736^T^(LC549121/1430)

**RG10^T^ (MZ148503/1406)**

*Geomonas terrae* Red111^T^ (MH915556/1426)

*Geomonas ferrireducens* S62^T^ (MH915555/1424)

*Geomonas edaphica* Red53^T^ (MH915554/1424)

*Geomonas oryzae* S43^T^ (MH915553/1424)

*Geomonas bremensis* Dfr1^T^ (U96917/1475)

*Geomonas limicola* Red745^T^ (LC505064/1433)

*Geomonas silvestris* Red330^T^ (LC505061/1426)

*Geomonas* *bemidjiensis* Bem^T^ (AY187307/1354)

*Desulfuromonas acetoxidans* DSM 684^T^ (AAEW02000008/1558)

*Geotalea* *uraniireducens* Rf4^T^ (EF527427/1359)

*Geotalea* *daltonii* FRC-32^T^ (EU660516/1424)

*Geotalea* *toluenoxydans* TMJ1^T^ (EU711072/1440)

*Geobacter* *luticola* OSK6^T^ (AB682759/1478)

99

99

96

99

85

74

97

74

100

99

98

81

100

100

84

72

0.01

**Supplementary Figure S2.** Neighbor-joining tree based on the 16S rRNA gene sequence of five strains*.* Bootstrap values (expressed as percentages of 1000 replications) greater than 50% are shown at branch points. Bar, 0.01 substitutions per nucleotide position. *Desulfuromonas acetoxidans* DSM 684^T^ is used as an outgroup. Number in the parenthesis is the GenBank accession number/ length of 16S rRNA gene (bp).

**RG29 (MZ148493)**

**RF4^T^ (MZ148489)**

**RG2^T^ (MZ148482)**

**RG3 (MZ266328)**

*Geomonas paludis* Red736^T^ (LC549121)

**RG10^T^ (MZ148503)**

*Geomonas terrae* Red111^T^ (MH915556)

*Geomonas ferrireducens* S62^T^ (MH915555)

*Geomonas edaphica* Red53^T^ (MH915554)

*Geomonas oryzae* S43^T^ (MH915553)

*Geomonas bremensis* Dfr1^T^ (U96917)

*Geomonas limicola* Red745^T^ (LC505064)

*Geomonas silvestris* Red330^T^ (LC505061)

*Geomonas* *bemidjiensis* Bem^T^ (AY187307)

*Desulfuromonas acetoxidans* DSM 684^T^ (AAEW02000008)

*Geotalea* *uraniireducens* Rf4^T^ (EF527427)

*Geotalea* *daltonii* FRC-32^T^ (EU660516)

*Geotalea* *toluenoxydans* TMJ1^T^ (EU711072)

*Geobacter* *luticola* OSK6^T^ (AB682759)

99

97

90

51

68

80

59

81

91

99

99

64

97

**Supplementary Figure S3.** Maximum-parsimony tree based on the 16S rRNA gene sequence of five strains*.* Bootstrap values (expressed as percentages of 1000 replications) greater than 50% are shown at branch points. *Desulfuromonas acetoxidans* DSM 684^T^ is used as an outgroup. Number in the parenthesis is the GenBank accession number.


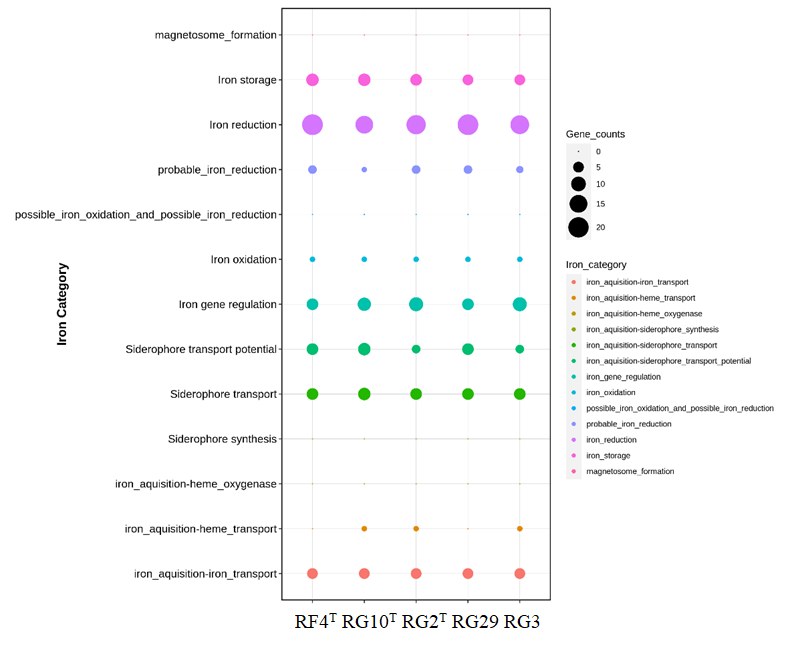


**Supplementary Figure S4:** Iron genes identified in the present study strains.


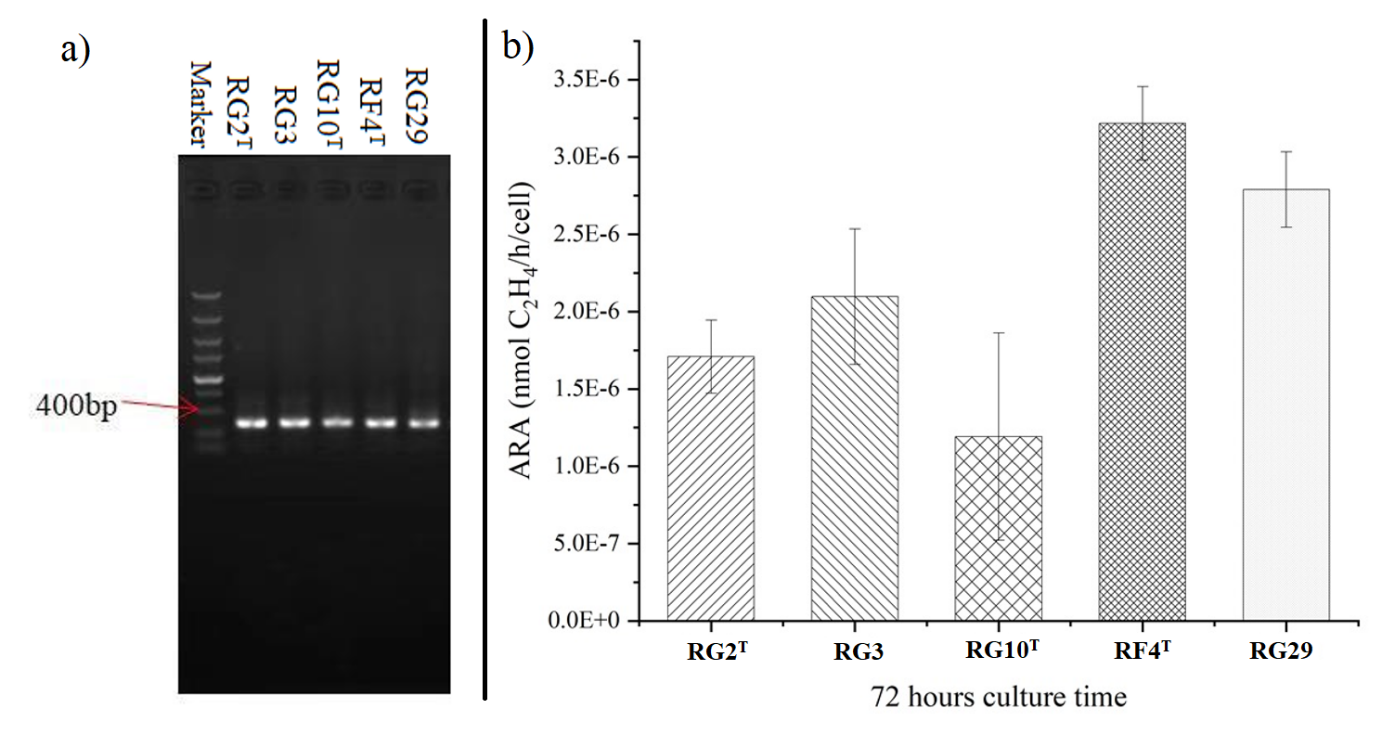


**Supplementary Figure S5.** Nitrogen fixation activity of five strains: a) PCR amplification of *nifH* gene; b) activity of C_2_H_2_ reduction into C_2_H_4_ by nitrogenase. All data presented were average standard deviations of triplicate.


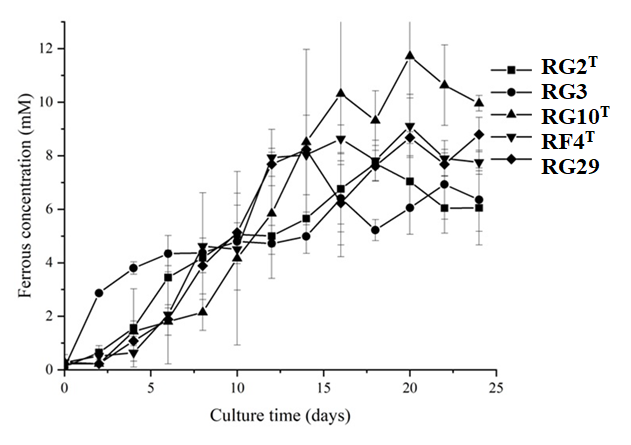


**Supplementary Figure S6.** Reduction of ferrihydrite of the five strains at different culture time with acetate as the electron donor. All data presented were average standard deviation of triplicate.

| 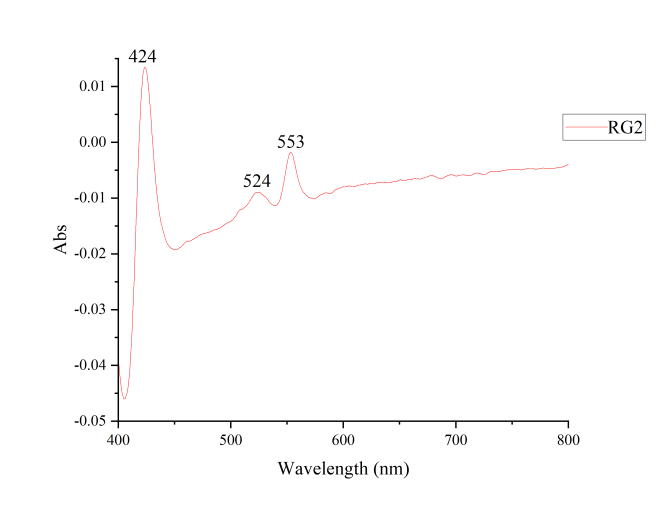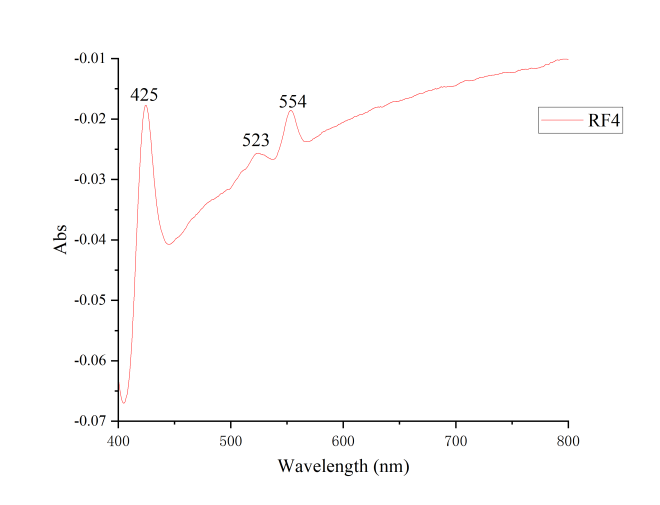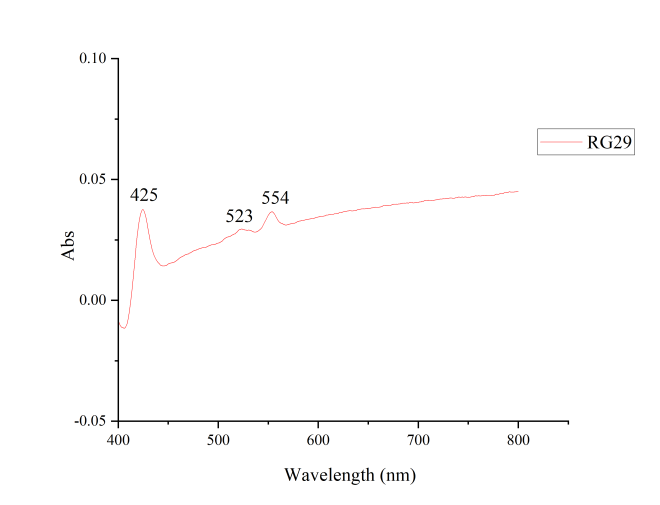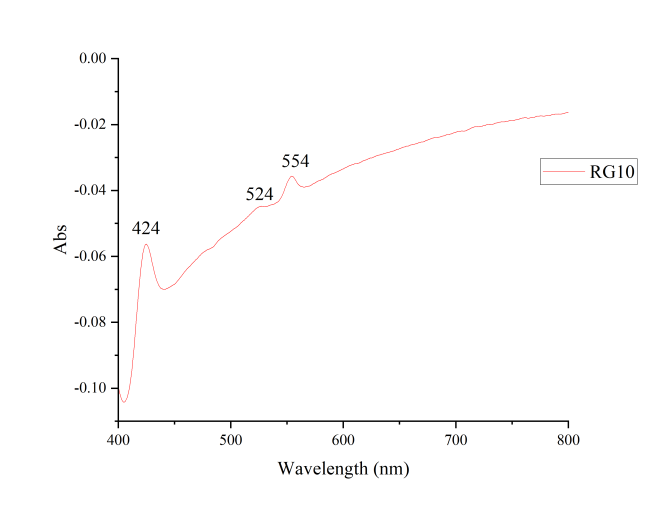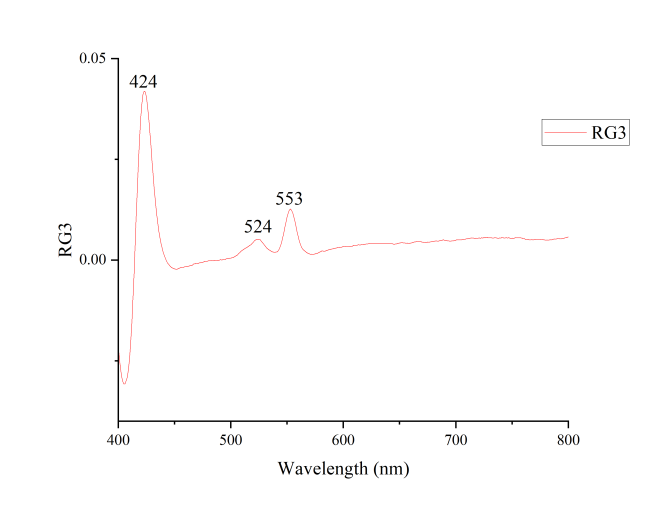 |
| --- |

**Supplementary Figure S7**. Different spectrum of whole cells for five novel strains in the wavelength range of 400–800 nm. The values shown in the pictures were wavelength of every peak.
